# Degradation of Binocular Coordination during Sleep Deprivation

**DOI:** 10.3389/fneur.2016.00090

**Published:** 2016-06-13

**Authors:** Jianliang Tong, Jun Maruta, Kristin J. Heaton, Alexis L. Maule, Umesh Rajashekar, Lisa A. Spielman, Jamshid Ghajar

**Affiliations:** ^1^Brain Trauma Foundation, New York, NY, USA; ^2^United States Army Research Institute of Environmental Medicine, Natick, MA, USA; ^3^Department of Environmental Health, Boston University School of Public Health, Boston, MA, USA; ^4^Department of Neurosurgery, Stanford University School of Medicine, Stanford, CA, USA

**Keywords:** alertness, attention, fatigue, ocular pursuit, screening

## Abstract

To aid a clear and unified visual perception while tracking a moving target, both eyes must be coordinated, so the image of the target falls on approximately corresponding areas of the fovea of each eye. The movements of the two eyes are decoupled during sleep, suggesting a role of arousal in regulating binocular coordination. While the absence of visual input during sleep may also contribute to binocular decoupling, sleepiness is a state of reduced arousal that still allows for visual input, providing a context within which the role of arousal in binocular coordination can be studied. We examined the effects of sleep deprivation on binocular coordination using a test paradigm that we previously showed to be sensitive to sleep deprivation. We quantified binocular coordination with the SD of the distance between left and right gaze positions on the screen. We also quantified the stability of conjugate gaze on the target, i.e., gaze–target synchronization, with the SD of the distance between the binocular average gaze and the target. Sleep deprivation degraded the stability of both binocular coordination and gaze–target synchronization, but between these two forms of gaze control the horizontal and vertical components were affected differently, suggesting that disconjugate and conjugate eye movements are under different regulation of attentional arousal. The prominent association found between sleep deprivation and degradation of binocular coordination in the horizontal direction may be used for a fit-for-duty assessment.

## Introduction

To aid a clear and unified visual perception while tracking a moving target, both eyes must be coordinated, so the image of the target falls on approximately corresponding areas of the fovea of each eye. The coupling between the two eyes can be algebraically described as the combination of the conjugate component, the degree to which the two eyes move in the same direction, and the disconjugate component, the degree to which the two eyes move in opposite directions. Generally, conjugate eye movements during tracking reflect the spatial angular displacement of the target projected on a fronto-parallel plane while horizontal disconjugacy varies with the visual depth.

Generating precise eye movements requires continual neural calibration through visual feedback, but eye movements generated in darkness in an alert state can be qualitatively similar to those generated during visual exploration of a static image, with scan paths consisting of series of saccades and fixations. In contrast, low arousal reduces the regulation of oculomotor control (1–3). During sleep, particularly during the rapid eye movement phase, the movements of the two eyes become uncoordinated (4). While the absence of visual input during sleep may also contribute to binocular decoupling, sleepiness is a state of reduced arousal that still allows for visual input. Sleep deprivation has significant effects on arousal (5), but has little effects on early visual processing such as contrast sensitivity and visual acuity (6, 7). Therefore, sleep deprivation provides a context within which the role of arousal in binocular coordination can be studied.

During sleep deprivation, peak velocity of saccades is reduced, and gaze–target synchronization of visual tracking is deteriorated (8–11). However, the effects of reduced arousal during wakefulness on binocular coordination are not well understood. Horne (12) examined visual functioning during 64-h sleep deprivation, during which the ability to maintain binocular convergence at both near and far visual distance decreased, but only after the first 24 h. Quant (13) also reported a decline in the ability to converge the eyes for binocular fusion with added horizontal prism power after 48 h of sleep deprivation, but other binocular functions such as convergence at near distance and stereopsis were clinically normal. Although these studies did not find degraded binocular coordination during an earlier stage of sleep deprivation, their methodologies were limited to measure the characteristics of binocular coordination as static changes. Recently, by continuously recording eye movements during simulated driving lasting for up to 1 h, Wakui and Hirata (14) detected a transient loss of the ability to maintain binocular coordination due to a transient reduction of arousal. Thus, monitoring the stability of binocular coordination may reveal performance degradation during an early stage of sleep deprivation.

We previously developed a standardized predictive visual tracking test procedure involving a continuous circular movement of a target, with which we have characterized degradations of monocular gaze–target synchronization during sleep deprivation (10, 11, 15, 16). Using the same two-dimensional predictive visual tracking protocol, we now examine how sleep deprivation affects the precision of binocular coordination. Specifically, we aim to characterize changes in the dynamics of two-dimensional coordination during acute one-night sleep deprivation. Such characterization may support early identification of fatigue-related performance decrements (16, 17).

## Materials and Methods

### Subjects and Testing Procedure

This study presents a new analysis of data previously published (10, 16). Military volunteers were tested at the United States Army Research Institute of Environmental Medicine (USARIEM), Natick Soldier Center, Natick, MA, USA. The experimental protocol was reviewed and approved by the USARIEM Institutional Review Board. Written informed consent was obtained from all subjects prior to data collection. Male and female subjects 18–50 years of age with at least 12 years of education were recruited. The subjects had no history of head injury with loss of consciousness, no substance abuse history, no known neurological disorders, no major psychiatric disorders, vision no worse than 20/30 after correction, and no reported hearing problems. Family history of psychiatric disorders was not assessed. Soldier medical readiness evaluations include eye exams as a routine element. Vision problems that could not be corrected constituted an exclusion criterion.

The subjects sustained wakefulness for a period of 26 h, during which measurements of visual tracking performance were taken at three time points. The study protocol was described in detail in previous publications (10, 16). Eighty-seven subjects completed the requisite sleep deprivation protocol, and their performance change was characterized in these publications using various metrics including smooth pursuit gain and phase error. Sleep on the night preceding the study was as per normal habit. The baseline measurement (Time 1) took place between 0630 and 0930 hours, which coincided with the subjects’ typical morning schedules. The second measurement (Time 2) was at predawn between 0200 and 0400 hours, and the last measurement (Time 3) was again in the morning between 0630 and 0930 hours. A member of the research team accompanied the subjects to ensure wakefulness throughout the entire experimental period, during which the subjects engaged in ordinary activities, including mild to moderate physical activity. Caffeine/stimulant consumption was prohibited during this period.

Among the 87 subjects who completed the sleep deprivation protocol, 52 subjects were also part of a descriptive study of the visual tracking task that included 2-week test–retest analyses (15). Using this subset, we conducted a pilot test–retest analysis on the metrics specific to this study. The visual tracking task and video-oculography procedures were described in detail in the previous publication (15). Briefly, testing was conducted in a well-illuminated room without a window to the outside, with the visual stimulus presented on a 120-Hz LCD monitor (Samsung SyncMaster 2233RZ) placed 47.5 cm in front of the subject whose head was stabilized by a head and chin rest. Binocular eye positions and pupil sizes were recorded at a sampling rate of 500 Hz with time-stamped target positions (EyeLink CL, SR Research, ON, Canada). The output was filtered using the device’s default setting (“Extra”). The test stimulus, presented twice, was a target that moved six times around a circle at a constant speed in the clockwise direction at 2.5 s per cycle against a black background. The circular trajectory had a radius of 300 pixels on the screen, corresponding to 10° visual angle. The target had the appearance of a red 0.5° diameter contour around a 0.2° black dot. Each visual tracking trial was preceded and succeeded by a period of central fixation, and two identical trials were administered consecutively. The entire testing sequence, which also included camera setup and calibration, lasted for approximately 5 min.

### Eye Movement Analysis

To study binocular coordination, it has traditionally been preferred that calibration to be conducted separately for each eye, although binocular calibration can yield similar results to monocular calibration (18). In this study, binocular calibration was justified because we examined relative changes in eye movement signals. Gaze position calibration was implemented using a nine-point fixation sequence under a binocular viewing condition and validated with a repeat fixation at each original target location. The calibration–validation records were used to select subjects whose average calibration–validation error of nine-point fixation was <1° in visual angle and maximum error within nine-point fixation <1.5° (“Good” validation, SR Research) for both eyes. Subjects whose calibration was not “Good” in either eye at any of the three testing time points were excluded from the data analysis. Recordings thus determined to be valid were available from 80 out of the 87 subjects. Among the subset of subjects who had completed the test–retest protocol, valid records were obtained from 46 subjects.

The calibration mapped the gaze of each eye on the screen. Pixel-based gaze and target coordinates were rescaled, so that a gaze displacement of 300 pixels from the center of the circular trajectory was matched by a 10° eye rotation, and the center of the circular trajectory of the target was represented by a 0° rotation. The resulting linearly scaled gaze representation was considered to approximate the angle of eye rotation from the gaze directed at the center of the target trajectory since within ±15°, eye rotation θ, re-expressed in radians, was approximately tan (θ).

Eye movement data were analyzed using custom MATLAB scripts (Matlab R2011b, MathWorks, Natick, MA, USA). The polarity of gaze position was defined as positive to the right in the horizontal direction and up in the vertical direction. The data from the first stimulus cycle of each of the two 6-cycle trials were not analyzed since the segment contained the initial transient response to the target movement. Thus, the data from a total of 10 stimulus cycles, corresponding to 25 s, were analyzed.

A partial occlusion of the eyes or other events that could produce incorrect gaze position information would also affect the pupil size records. The quality of gaze position records was examined by checking the concurrent pupil size records, and comparing them with the measurement obtained from a 2-mm hole in a plate, which simulated a pupil. The pupil diameter is normally no smaller than 2 mm (19), and the size of 2 mm pupil corresponded to 750 U recorded on our EyeLink eye tracker setup. Therefore, any sample associated with a pupil size <600 U, i.e., 80% of a 2-mm pupil in our setup, was attributed to a semi-blink or other partial occlusion of the eye and was discarded. The pupil image is elliptical and its contour eccentricity modulates in relation to eye position. The pupil size record should modulate accordingly, and a rapid change would likely be associated with artifacts. Thus, data segments containing a pupil size change exceeding an equivalent to a pupil constriction speed of 6 mm/s were discarded (20). Horizontal and vertical eye velocities were computed by two-point differentiation of the position data. Magnitude of the eye velocity vector over 1000°/s was considered to be physiologically impossible (21), and such data segments along with the neighboring 200 ms were also discarded.

The horizontal and vertical *disconjugate components* of binocular visual tracking were calculated as left − right gaze positions. To characterize the stability of binocular coordination, their SDs (SDHDC and SDVDC for horizontal and vertical, respectively) were computed over all valid samples within the 25-s period of data. Smaller values of SDHDC and SDVDC indicate a better dynamic control of binocular coordination in the horizontal and vertical directions, respectively. A pilot analysis of test–retest intraclass correlation (ICC) with one-way random effect model (22) of log transformed SDHDC and SDVDC yielded 0.48 and 0.38, respectively, indicating fair reliability. Thus, changes in these metrics during sleep deprivation, with the Time 1 measurement serving as a baseline control, should demonstrate effects of sleep deprivation.

The horizontal and vertical *conjugate components* of binocular visual tracking were calculated as the averages of the left and right gaze positions. Horizontal and vertical conjugate errors were defined as the differences between the conjugate gaze positions and the target positions. To characterize the stability of the conjugate gaze relative to the target, we calculated their SDs over all valid samples within the 25-s period of data (SDHC and SDVC for horizontal and vertical, respectively). Smaller values of SDHC and SDVC indicate a better dynamic control of gaze–target synchronization in the horizontal and vertical directions, respectively. The test–retest ICCs of log transformed SDHC and SDVC were 0.69 and 0.72, respectively, indicating substantial reliability. Thus, changes in these metrics during sleep deprivation, with the Time 1 measurement serving as a baseline control, should demonstrate effects of sleep deprivation.

### Statistical Analysis

Statistical analyses were conducted using IBM SPSS V.20 (IBM Corp., Armonk, NY, USA). Changes in calibration and visual tracking performance across the three time points (baseline, predawn, and 26 h) were examined with a mixed-effects linear model designed to detect the subject-level pattern of change by time, by the directions of eye movements (horizontal or vertical), and by their interaction. This approach accounts for within-subject dependence in the data and between-subject variability. As planned comparisons, within group changes by time were examined between pairs of time points. A receiver operating characteristic (ROC) analysis was used to examine the group-wise changes in gaze–target synchronization and binocular coordination from Time 1 to Time 3, assuming the baseline Time 1 measures as normal and the Time 3 measures as abnormal and testing for the correctness of binary classification with the cut-point for discrimination sliding across the data range. A larger area under the ROC curve indicates a better distinction of oculomotor performance associated with sleep deprivation. All tests were evaluated at a 0.05 significance level.

## Results

### Oculomotor Control during Visual Tracking

**Figure 1:**
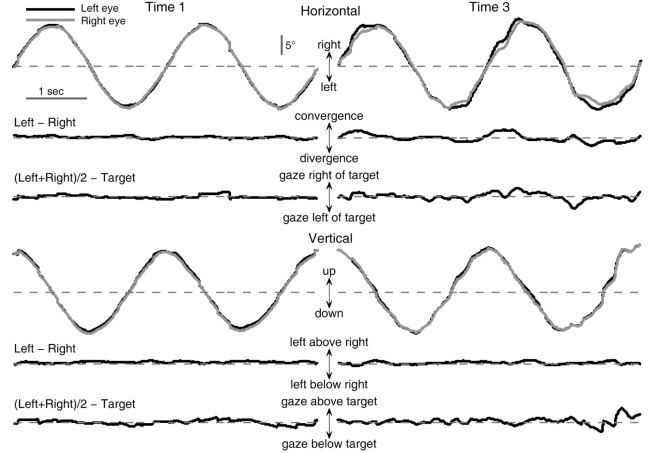
**Effects of sleep deprivation on circular visual tracking performance of a typical subject (033)**. Left column: baseline performance (Time 1). Right column: performance at 26 h of sleep deprivation (Time 3). Shown are excerpts from the last two cycles of the first six-cycle trial of the respective recording session.

Examples of binocular eye movement traces during tracking of a circular target movement are shown in Figure 1. The left and right columns show performance at baseline and after one night of sleep deprivation, respectively. The top traces in each panel are raw positions of the left and right gaze as a function of time during two cycles of circular movement of the target starting from the 12 o’clock position. The two sets of traces shown below are the differences between the positions of the left and right eyes (“Left − Right”) and between the averaged eye position [“(Left + Right)/2”] and the target. The deviations from the dashed horizontal line indicate mismatches between the left and right gaze positions or between the average gaze and the target. Compared with baseline performance (left column), performance after one night of sleep deprivation (right column) was characterized by increased variability in mismatches between the left and right gaze positions as well as between the average gaze and the target in both horizontal and vertical directions. These observations justified the use of variability metrics in characterizing performance changes.

**Figure 2:**
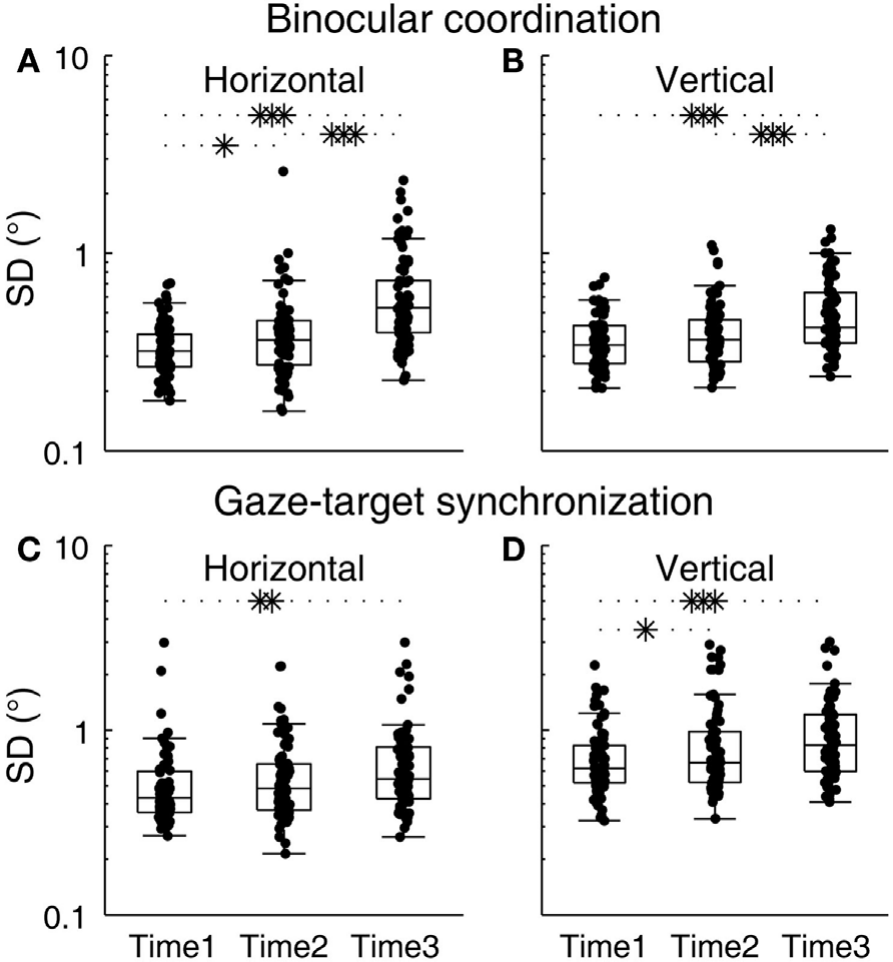
**Changes in binocular coordination and gaze–target synchronization during the course of sleep deprivation**. **(A)** SDHDC, **(B)** SDVDC, **(C)** SDHC, and **(D)** SDVC. Box plots are shown with individual scores. The upper and lower hinges of each box represent the 75th and 25th percentiles, respectively. The horizontal inside the box represents the median of the scores. The upper and lower whiskers extend to the maximum and minimum of 1.5 times the interquartile range. *p < 0.05, **p < 0.01, ***p < 0.001, pairwise comparison of outcomes at different time points against the null hypothesis that outcomes were no different.

Changes in binocular coordination during the sleep deprivation period were examined (Figures 2A,B). The group averages of SDHDC at Times 1, 2, and 3, were 0.34, 0.42, and 0.66, respectively, and those of SDVDC were 0.37, 0.40, and 0.52, respectively. The subject-level change across the testing time points was significant [*F*_(2, 196.183)_ = 34.442, *p* < 0.0001]. An overall difference between horizontal and vertical binocular coordination was statistically not significant [*F*_(1, 259.015)_ = 3.738, *p* = 0.054], but there was a significant time–direction interaction [*F*_(2, 196.183)_ = 4.62, *p* = 0.011]. SDHDC and SDVDC were not significantly different from each other at Time 1 [*t*_(157.663)_ = −1.499, *p* = 0.136] or Time 2 [*t*_(127.991)_ = 0.481, *p* = 0.631], but were different at Time 3 [*t*_(123.841)_ = 2.623, *p* = 0.01], with SDHDC larger than SDVDC. For the changes within each direction, planned comparisons revealed that significant changes took place between each pair of time points in SDHDC [Times 1 and 3, *t*_(90.024)_ = −6.482, *p* < 0.0001; Times 2 and 3, *t*_(142.375)_ = −4.078, *p* < 0.0001; Times 1 and 2, *t*_(100.632)_ = −2.255, *p* = 0.026]. The changes in SDVDC were significant between Times 1 and 3 [*t*_(115.788)_ = −5.018, *p* < 0.0001] and Time 2 and 3 [*t*_(146.403)_ = −3.430, *p* = 0.001], but not between Times 1 and 2 [*t*_(137.295)_ = −1.468, *p* = 0.145].

Changes in gaze–target synchronization during the sleep deprivation period were examined next (Figures 2C,D). The group averages of SDHC at Times 1, 2, and 3, were 0.53, 0.60, and 0.70, respectively, and those of SDVC were 0.73, 0.89, and 0.98, respectively. The subject-level change across the testing time points was significant [*F*_(2, 285.919)_ = 9.28, *p* < 0.0001], and there was an overall difference between horizontal and vertical gaze–target synchronization [*F*_(1, 409.23)_ = 40.235, *p* < 0.0001], with SDHC generally smaller than SDVC. There was no significant interaction between time and direction [*F*_(2, 285.919)_ = 0.605, *p* = 0.547]. Planned comparisons revealed that significant changes took place in SDHC between Times 1 and 3 [*t*_(151.061)_ = −2.474, *p* = 0.014], but not between Times 2 and 3 [*t*_(149.674)_ = −1.511, *p* = 0.133] nor between Times 1 and 2 [*t*_(151.919)_ = −1.113, *p* = 0.267]. The changes in SDVC were significant between Times 1 and 3 [*t*_(136.649)_ = −3.451, *p* = 0.001] and Times 1 and 2 [*t*_(129.016)_ = −2.098, *p* = 0.038], but not between Times 2 and 3 [*t*_(156.532)_ = −0.966, *p* = 0.335].

**Figure 3:**
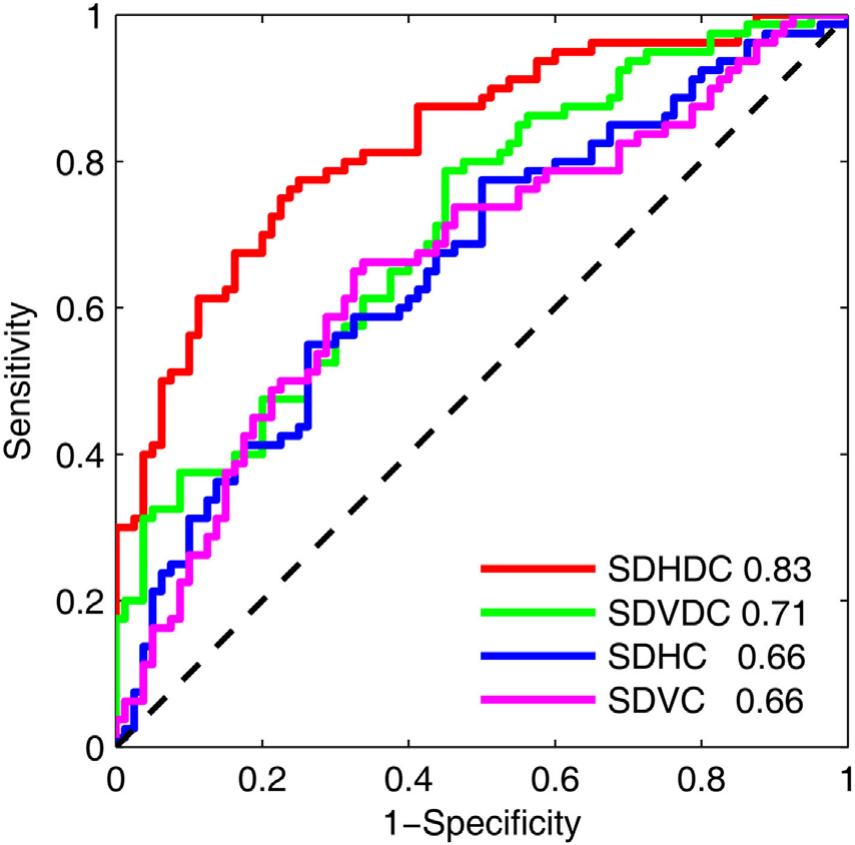
**Receiver operating characteristic of the effects of sleep deprivation on the binocular coordination and gaze–target synchronization in the horizontal and vertical directions**. The analysis was based on the results at Time 1 and Time 3. The area under each curve is shown.

The effectiveness of the metrics of gaze–target synchronization and binocular coordination to classify a sleep-deprived state was tested with an ROC analysis of binocular coordination and gaze–target synchronization at Time 1 and Time 3 (Figure 3). In this analysis, the samples from the two states were considered to be independent and within-individual changes were not accounted for. The areas under the curve were 0.83 for SDHDC, 0.71 for SDVDC, 0.66 for SDHC, and 0.66 for SDVC.

## Discussion

In this study, one night of sleep deprivation resulted in degraded stability of binocular coordination as measured by a visual tracking task. These findings support our propositions that a degradation of binocular coordination during sleep deprivation may occur earlier than previously reported (12, 13) and that it may be detected by analyzing the dynamics of binocular movements. The parameters of disconjugacy variability achieved a large area under the ROC curve, suggesting that the characterization of binocular coordination is well suited to complement those of gaze–target synchronization in the detection of detrimental effects associated with acute sleep deprivation.

There may be other explanations than reduced control of binocular coupling for changes in the disconjugacy variability metrics. Since saccades induce changes in vergence angle (23, 24), it is possible that a change in the frequency of saccades during sleep deprivation contributed to increased disconjugacy variability. Our previous analysis using monocular eye movement data showed that, indeed, sleep-deprived subjects made one additional saccade per 2.5 s cycle on average (an increase to 7.3 per cycle from 6.0 at baseline). However, this increase in the saccade rate was essentially ascribable to those with amplitudes smaller than 1° (10). Since the transient vergence associated with such small saccades should not exceed 0.1° per saccade incidence (23, 24), the changes in the saccade rate during sleep deprivation likely contributed little to the metrics of overall disconjugacy variability. Another possibility is that a change in accommodative function increased disconjugacy variability by reducing clarity of the target image. This alternate explanation is also unlikely to stand since reduced image clarity would be similar for both the horizontal and vertical direction and cannot account for our finding of the horizontal and vertical directional difference in the time courses of disconjugacy variability change.

Previously, we showed that the central nervous system monitors the temporal lag of the gaze relative to the target and compensates for an increase in the lag by increasing the rate of small anticipatory saccades during sleep deprivation (10). The results suggest that a monitoring or compensatory mechanism may be less effective for the control of binocular coordination. A possible explanation for this reduced efficiency is a relative tolerance for binocular disparity since humans can maintain perceptual binocular fusion outside the foveola with disparity as large as 2° (25), albeit still well within the 5°–7° diameter of foveal vision (26, 27). In addition, sensitivity for the binocular disparity may be reduced during visual tracking since stereothresholds significantly increase when smooth pursuit velocity exceeds 2°/s (28).

Visual tracking is normally more accurate in the horizontal than in the vertical direction (15, 29–31). The directional difference in performance suggests differences in the neural implementation of the controls of horizontal and vertical tracking: sleep deprivation appears to affect this dynamic differentially. Previously, in this same cohort of subjects, we found a clearer sleep deprivation-induced reduction of smooth pursuit velocity gain in the horizontal than in the vertical direction (10, 11). Presently, we found that while the positional precisions of gaze–target synchronization were degraded similarly between the two directions during sleep deprivation, the degradation in the precision of binocular control was asymmetrical, worse in the horizontal than in the vertical direction.

The relationships between the horizontal and vertical components of binocular coordination and gaze–target synchronization are altered differently to decrements in arousal indicates that binocular coordination and gaze–target synchronization are under different neural regulation of arousal. The high-level spatio-temporal planning of multi-dimensional visual tracking must be coded in a conjugate manner, but at some premotor level in the brain stem, eye positions are encoded monocularly (32, 33). The loss of binocular coordination can be explained as noise separately injected into the monocular commands of each eye. Given that the extent of disconjugacy during sleep is similar between the horizontal and vertical directions (4), what caused the more severe impact on horizontal binocular coordination in our sample is not clear. The neural circuitry responsible for the horizontal direction is equipped with more flexibility than that for the vertical direction to allow for visually guided learning related to depth perception (34, 35). A downside of this flexibility may be susceptibility for destabilization when drowsy.

In conclusion, sleep deprivation degrades the stability of both binocular coordination and gaze–target synchronization. Specifically, the prominent association found between sleep deprivation and degradation of binocular coordination in the horizontal direction may be utilized in a fit-for-duty assessment. However, there are technical challenges. Even though eye movements can be recorded rather easily and precisely with a video-based method, measuring and analyzing binocular data require special caution, without which the reliability of derived metrics may be reduced (36). High quality images from the two eyes must be obtained synchronously and continuously. The data must be carefully screened since a loss of the software or hardware’s ability to track one of the eyes immediately invalidates the binocularity of the sample. Since poor calibration of either eye alone can be mistaken for poor binocular coordination, valid calibration procedures are critically important. Technological improvement in eye tracking addressing challenges associated with binocular recording are much welcomed.

## Author Contributions

JM, KH, and JG designed experiments. AM and KH collected data. JT, JM, UR, and LS designed and conducted analyses. All authors contributed to the interpretation of data and to drafting and revising the work.

## Conflict of Interest Statement

JG is director of Sync-Think, Inc. and holds U.S. patent 7,384,399. JM holds stock option in Sync-Think. JG, JT, and JM have pending patent application PCT/US2014/050774 related to the subject matter described in this paper. The remaining co-authors declare that the research was conducted in the absence of any commercial or financial relationships that could be construed as a potential conflict of interest.
